# The Influence of Neighbourhoods and the Social Environment on Sedentary Behaviour in Older Adults in Three Prospective Cohorts

**DOI:** 10.3390/ijerph14060557

**Published:** 2017-05-24

**Authors:** Richard J. Shaw, Iva Čukić, Ian J. Deary, Catharine R. Gale, Sebastien F. M. Chastin, Philippa M. Dall, Manon L. Dontje, Dawn A. Skelton, Laura Macdonald, Geoff Der

**Affiliations:** 1 MRC/CSO Social and Public Health Sciences Unit, University of Glasgow, Glasgow G2 3QB, UK; laura.macdonald@glasgow.ac.uk (L.M.); geoff.der@glasgow.ac.uk (G.D.); 2 Department of Psychology Centre for Cognitive Ageing & Cognitive Epidemiology, University of Edinburgh, Edinburgh EH8 9JZ, UK; iva.cukic@ed.ac.uk (I.Č.); i.deary@ed.ac.uk (I.J.D.); cgale@staffmail.ed.ac.uk (C.R.G.); 3 MRC Lifecourse Epidemiology Unit, University of Southampton, Southampton S016 6YD, UK; 4 Institute for Applied Health Research, School of Health and Life Sciences, Glasgow Caledonian University, Glasgow G4 0BA, UK; sebastien.chastin@gcu.ac.uk (S.F.M.C.); philippa.dall@gcu.ac.uk (P.M.D.); dr.m.l.dontje@gmail.com (M.L.D.); dawn.skelton@gcu.ac.uk (D.A.S.); 5 Department of Movement and Sports Sciences, Faculty of Medicine and Health Science, Ghent University, Ghent 9000, Belgium; 6 School of Population and Global Health, University of Western Australia, Perth 6009, Australia

**Keywords:** sedentary behaviour, social environment, physical environment, ageing, health, neighbourhood, social capital, social support

## Abstract

Sedentary behaviour is an emerging risk factor for poor health. This study aimed to identify ecological determinants of sedentary behaviour, for which evidence is currently scarce. The study participants were community dwelling adults from, respectively, the Lothian Birth Cohort 1936 (n = 271, mean age 79) and the 1930s (n = 119, mean age 83) and 1950s (n = 310, mean age 64) cohorts of the West of Scotland Twenty-07 study. The outcome measure, percentage of waking time spent sedentary (sedentary time), was measured using an activPAL activity monitor worn continuously for seven days. Potential determinants included objective and subjective neighbourhood measures such as natural space, crime, social cohesion and fear of crime. Other determinants included measures of social participation such as social support, social group membership and providing care. Results from multivariable regression analyses indicated that providing care was associated with reduced sedentary time in retired participants in all cohorts. Fear of crime and perceived absence of services were associated with increased sedentary time for retired 1950s cohort members. Higher crime rates were associated with increased sedentary time in all cohorts but this was not significant after adjustment for socio-demographic characteristics. Most other neighbourhood and social participation measures showed no association with sedentary time.

## 1. Introduction

Sedentary behaviour, defined as energy expenditure ≤1.5 metabolic equivalents (METs) while awake and in a sitting or reclining posture [1], is emerging as a potentially modifiable risk factor for poor health [2]. There is evidence that sedentary behaviour is associated with increased risk of mortality [3,4,5], diabetes [6], cancer incidence [5], falls [7] and reduced bone density [8]. Sedentary behaviour increases with age [9]. On average, sedentary time represents 65–80% of an older adult’s waking day [10] and 67% of older adults spend in excess of 8.5 h per day sitting [11]. Reducing sedentary behaviour may lead to health improvements for older adults.

Understanding what determines sedentary behaviour in older adults is crucial for developing effective interventions. In order to understand the determinants of sedentary behaviour it is important to take an ecological approach and account for the physical and social context in which this behaviour occurs [2]. Owen and co-workers’ ecological model posits that the neighbourhood environment, including the physical and social environment in which people live and how they perceive it, are important determinants of sedentary behaviour [2]. In addition, the Systems of Sedentary Behaviours (SOS) framework created by the DEDIPAC (DEterminants of DIet and Physical ACtivity) consortium [12] highlights social and cultural settings, such as community activities and the influence of peers, and the built and natural environment as key priorities for research. Qualitative research supports this, with the outdoor environment, gardening, volunteering and socialising with people being identified as themes that encourage people to stand and be active [13,14]. However, there are few quantitative studies investigating the aspects of the social and environmental context that are important for sedentary behaviour in older adults [9].

We are aware of only three studies that have investigated relationships between objectively measured sedentary behaviour and aspects of the ecological environment in older adults [15,16,17]. Van Holle et al. [16] investigated sedentary behaviour’s associations with social cohesion, social diversity, and talking among neighbours for adults aged over 65 living in Ghent, Belgium. While no direct associations were found, there was an interaction between talking to neighbours and walkability, which reflects a neighbourhood’s convenience for transport walking. Talking to neighbours was associated with reduced overall sedentary time but only for neighbourhoods with high walkability. Sartini et al. [15] found no evidence of an association between sedentary behaviour and a measure of social isolation for older British men. Van Der Berg et al. [17] found that living in an apartment compared to a villa was associated with increased sedentary time after adjusting for health related factors for older people living in Iceland. These studies used ActiGraph accelerometers which measure lack of movement, which has known limitations [18], rather than posture.

Van Holle et al., along with five additional studies, investigated relationships between ecological factors and sedentary behaviour in older adults, with sedentary behaviour operationalized using self-reported measures of sitting time or TV watching [16,19,20,21,22,23]. The potential ecological determinants of sedentary behaviour included aspects of the physical environment such as access to shops, pedestrian infrastructure, attractiveness of the neighbourhood [19,20,21,22], and self-reported measures of the presence of greenery or parks [19,22]. Aspects of social environment included area influences such as fear of crime [20,22], social cohesion or neighbourhood attachment [19,20], and individual specific interactions such as volunteering [19], participation in social or community groups or talking to neighbours [16,19,20]. Overall, these studies did not show consistent associations and this may in part be because self-reported measures of sedentary behaviour have only low to moderate validity [24,25] and, therefore, attenuate the true strength of associations.

Alternatively, results may vary across studies because older adults are not a homogeneous group. For example, one study found that a reduction in self-reported TV viewing was associated with increased social cohesion and perceived safety for retired people but did not find the same relationship for employed people [20]. It is also possible that the influence of ecological determinants on sedentary behaviour vary among retired people. It has been argued that there is a period of early old age (65–74), which has been termed the Third Age, where people are freed from the constraints and restrictions of employment but have a much lower risk of the constraints of infirmity and poverty than at older ages [26]. Thus, they may have a greater sense of agency and freedom to pursue leisure activities and, consequently, the social and physical environment is likely to be more influential.

In summary, few studies have investigated the socio-ecological determinants of sedentary behaviour in general, and studies using objective measures of sedentary behaviour are a tiny proportion of these. Many important ecological risk factors have only been investigated in one or two studies or not at all, and results in this limited literature vary between studies, and possibly by age and retirement status. In this study, we aim to investigate which aspects of the neighbourhood and social environment predict objectively measured sedentary behaviour in three cohorts of older adults, using a more comprehensive range of subjective and objective measures than previous studies [9].

## 2. Materials and Methods

This study, Seniors USP (Understanding Sedentary Patterns), comprises subsamples of the Lothian Birth Cohort 1936 (LBC1936) and the West of Scotland Twenty-07 study (Twenty-07). Full details for these studies are available elsewhere [27,28]. The Twenty-07 study itself comprises three age cohorts, although only the two oldest are included here (hereafter referred to as the 1930s and 1950s cohorts according to their decade of birth). Data for the main study were collected in five waves of interviews between 1987, when the 1950s and 1930s cohorts had respectively a mean age of 36 years and 56 years, and 2008. LBC1936 is an on-going cohort study that began in 2004, when participants were 69 years old, as a follow up to the Scottish Mental Survey 1947.

Data specific to this study, including objective sedentary behaviour, were collected between November 2014 and April 2016. The 1930s cohort (mean age 83 years) and 1950s cohort (mean age 64 years) were interviewed in their own homes by trained nurses, while LBC1936 (mean age 79 years) participants were interviewed at a clinical research facility by psychology graduates and post docs. In addition, we drew data from wave 1 (2004 to 2007) and wave 2 (2007 to 2010) of LBC1936 and wave 5 (2007/2008) of Twenty-07.

To be eligible, participants needed sufficient cognitive ability to be able to provide informed consent and complete sleep diaries during the period in which they wore activity monitors. Beyond these minimum requirements, people were not excluded due to physical or mental impairments. Twenty-07 participants were eligible if they lived within the Greater Glasgow area. All eligible people in the 1930s cohort were approached and a random sample of eligible people in the 1950s cohort was selected. Consecutive recruits to wave 4 of LBC1936 were invited to join Seniors USP until the target sample size was achieved. All participants provided written informed consent. Ethics approval for the Twenty-07 West of Scotland study was obtained from the National Health Service and/or Glasgow University Ethics Committees. Ethical approval for LBC1936 was obtained from the Multi-Centre Research Ethics Committee for Scotland.

### 2.1. Sedentary Behaviour

The outcome measure was the percentage of waking time spent sedentary, averaged over the seven days (hereafter, sedentary time). Waking time was derived from diaries. Sedentary behaviour was measured using the activPAL monitor (activPAL3c, PAL Technologies Ltd., Glasgow, UK) which provides accurate and reliable measurement of sedentary behaviour [29,30]. The device is a small and light (53 × 35 × 7 mm; 15 g) tri-axial inclinometer. It is worn attached to the anterior thigh of the dominant leg with a waterproof dressing and continuously monitors the position of the thigh. Participants were initially interviewed for basic socio-demographic and health information and were then asked to wear the activPAL continuously for seven days, including overnight and during bathing/swimming, while going about their usual daily activities. Participants also kept a diary reporting the time they fell asleep the previous night and the time they woke up for each day of monitoring.

### 2.2. Independent Variables

Independent variables were classified into 5 different categories: objective neighbourhood, subjective neighbourhood, social support, social participation, and home environment measures.

#### 2.2.1. Objective Neighbourhood Measures

The neighbourhood environment has been operationalized objectively by linking participants’ postcode of residence at the time of Seniors USP (2014 to 2016) to Scottish Government data zones and census output areas. Data zones are the key small-area statistical geography in Scotland and contain populations of between 500 and 1000 people. The measures we have at this level, and described in more detail below, are natural space, Scottish Index of Multiple Derivation (SIMD) 2012 Access domain, SIMD Crime domain, walkability, pensioner density and population density. In addition, we have green space data available at census output area [31].

Percentage of natural space for each data zone was calculated from Scotland’s Greenspace Map; obtained from Central Scotland Green Network (CSGN), which covers settlements with populations greater than 3000 and refers to land cover in 2011. The natural space measure includes diverse area types including parks, woodland, playing fields, agricultural land, school grounds, bowling greens and open water including lakes, river or canals. However, manmade surfaces such as tennis courts and squares were excluded.

We used two domains from SIMD to indicate crime and access to services [32]. The SIMD crime domain is based on recorded crime rates for data zones for the following crimes: crimes of violence, sexual offences, domestic housebreaking, vandalism, drugs offences and common assault. The access to services measures includes travel time (driving and using public transport) to access basic services such as General Practice Surgeries, Post Offices, schools and retail centres. For both measures fractional ranks have been calculated for Scotland, where data zones have been ranked for each deprivation domain and divided by the total number of data zones, with higher ranks indicating disadvantage. These measures can be considered a slope index of inequality (SII) [33] and represent the difference between lowest and high crime rate areas, or the most versus the least accessible areas.

We used a two component measure of walkability of the local area [34]. The first component is dwelling density, which is the ratio of residential units to land area [35]. High dwelling density areas tend to become less car dependent (e.g., it is more difficult to drive and park) and more convenient for walking. The second component, intersection density, is derived from the street network dataset and path network data set for Scotland, both for 2011 [36,37]. When intersection densities are high, the route between origin and destination is more direct and quicker. The walkability score is calculated as: (2 × intersection density z score) + (dwelling density z score). Intersection density was weighted by two as previous work highlights the strong influence of this measure on active travel choices [38].

Two neighbourhood measures were taken from the 2011 Scottish census. First pensioner density was operationalized as the percentage of people aged over 65 within the data zone. The proportion of people over 65 might influence the availability of formal services, voluntary and community groups and the ability to form informal networks with people of a similar age. Second, population density (number of persons per hectare) may reflect the nature of the physical environment in which people live.

In addition, for participants living within the Glasgow and Edinburgh 2011 Census Travel to work areas, percentage green space was measured at output area. The percentage green space was defined as the percentage of the total area that was either Forest or Green Urban Areas using data from the European Environment Agency Urban Atlas.

#### 2.2.2. Subjective Neighbourhood Measures

We included 6 measures of participants’ subjective views of their neighbourhoods collected in wave five of Twenty-07 between 2007 and 2008.

Social cohesion [39] comprised five items, e.g., “This is a close-knit neighbourhood”, rated on a five point Likert scale with greater scores indicating greater neighbourhood cohesion.

Neighbourhood problems have been assessed using three measures from the Twenty-07 study. Participants were asked to rate 16 different problems in their neighbourhood on a three point scale. Prior research has shown these items are related to three distinct domains [40], incivilities, (e.g., litter, vandalism, and burglaries); absence of goods (e.g., difficulties obtaining services, and lack of recreation facilities); and physical environmental problems (e.g., uneven/dangerous pavements, speeding traffic and waste ground). For each domain, a score was constructed by summing the items making up the domains.

Fear of crime was assessed for Twenty-07 participants with a widely used question [41,42] asking how people feel about walking around the area after dark with the responses being; never do it under any circumstances, try to avoid doing it, do it but feel uncomfortable, have no worries about doing it. Feelings about living in the area were assessed using a visual scale with seven faces ranging from very happy to very sad [43].

For the LBC1936 cohort an 8 item neighbourhood attachment scale was used with data being collected in wave 2. Participants were asked to rate their agreement with five items, e.g., “I feel like I belong to this neighbourhood”, on a five point Likert scale [44,45].

#### 2.2.3. Social Participation Measures

In wave 5 of Twenty-07 (with a reference to a period of four weeks), and wave 1 of LBC1936 (with a reference to a period of two weeks) participants were asked if they had been in contact with people, excluding those they lived with. Forms of contact included chatting with a family member, chatting with a friend, contact by letter, telephone or email with a family member, and contact by letter, telephone or email with a friend. This was summed to form a continuous measure scoring from zero to four.

Twenty-07 participants in wave 5 were asked whether or not they regularly participated in the activities of different types of organization. Types of organization included: church, religious groups or charitable organisations; education, (e.g., art groups, music groups or evening classes); social clubs (e.g., rotary club, women’s institute, Townswomen’s Guild, working men’s clubs or elderly lunch groups); and sports groups (e.g., sports clubs, gym or exercise classes).

#### 2.2.4. Social Support Measures

Two measures of social support from each of LBC1936 and Twenty-07 were available: satisfaction with social support and perceived social support for LBC1936; emotional support and practical support for Twenty-07.

The LBC1936 wave 1 measure of satisfaction with social support comprises 12 items (e.g., “How often were there people who you could really count on to be dependable when you needed help?”), answered on a five point scale (from “all of the time” to “none of the time”).

Perceived social support was assessed at wave 2 of LBC1936 with a scale previously used in the Health Survey for England [46]. Respondents were asked to indicate if each of 7 items were not true, partially true, certainly true, e.g., “There are people I know amongst my family or friends who do things to make me feel happy”. The items were summed to form a continuous scale.

In wave 5 of Twenty-07, there was one measure of emotional support which asks “Are there other people you could talk to about your problems and share your worries with?” and “If yes: about how many people would you share your problems with?” In addition, there was a question asking for practical support “If you had a serious problem, perhaps like an illness which meant you had to stay in bed for a week or more, is there someone you could turn to for practical help?”, if they said yes they were asked how many people they could ask for practical help.

#### 2.2.5. Home Environment

Twenty-07 participants were allocated into two groups based on their garden status in wave 5 (own garden or backyard/other). In addition, interviewers completed responses to type of accommodation (detached house/semi-detached/terraced/all types of flats and other combined), presence of internal stairs (one level/with stairs), what type of entry (ground floor/all other floors).

### 2.3. Statistical Methodology

Following prior research [20] including our own study [47] which suggests that the influence of the social environment on sedentary behaviour may differ before and after retirement, we have divided the 1950s cohort into those still employed, including the semi-retired, versus those no longer employed. We refer to the latter as “retired” even though not all would consider themselves formally retired. Analyses are presented for the cohorts separately and for retired people in the 1950s cohort combined with people from the Twenty-07 1930s cohort and LBC1936 cohorts (hereafter referred to the as the combined retired group). In the latter analyses, we adjusted for cohort. The main analyses were conducted using simple linear regression and then multivariable regression investigating each independent variable of interest separately but adjusting for the following potentially confounding factors marital status at time of Seniors USP (Married/Cohabiting/Single/Divorced/Separated, Widowed), gender, education (No formal qualifications/Basic e.g., O-levels, A-levels or equivalents/Advanced e.g., degree or professional qualification) and the Carstairs measure of area deprivation based on the 2011 census [48,49]. Carstairs deprivation is used as it does not include the same indicators as the SIMD access and crime domains and we have shown this measure of socioeconomic position to be associated with sedentary behaviour using data from this study [47]. All analyses were conducted using Stata version 13.1.

## 3. Results

Seven hundred and seventy three participants took part: 340, 129, and 304 each from the 1950s cohort, 1930s cohort, and LBC1936, respectively. Of these 700 (91%) provided seven full days of activPAL and sleep diary data.

### 3.1. Descriptive Statistics

Continuous measures by cohort and employment status are shown in Table 1 and categorical measures are shown in Table 2 and Table 3. As we have reported previously [47], the 1930s cohort were the most sedentary (68%), the 1950s employed cohort the least (58%), and retired people in the other two cohorts were in between (62%). For measures that were available for both LBC1936 and the Twenty-07 cohorts, LBC1936 were generally more advantaged with the exception of natural and green space measures for which the Twenty-07 cohorts had higher averages. For measures that were only available for the Twenty-07 study, the 1950s employed cohort tended to be more advantaged than the 1950s retired cohort and the 1930s cohort.

### 3.2. Determinants of Sedentary Behaviour

#### 3.2.1. Objective Neighbourhood Measures

The associations between sedentary time and objective neighbourhood measures, both before and after adjustment for marital status, gender, education and Carstairs deprivation, are shown in Table 4. Even in unadjusted analyses, there are few significant associations, and regression coefficients for the 1950s employed cohort are generally smaller than for the other groups. SIMD crime was the only measure to show a consistent relationship with sedentary time, and this association was dramatically reduced on adjustment. As an illustration, for the combined retired group, the difference in sedentary time between those living in the highest crime areas compared to those in the lowest crime areas decreases from 6.29% higher sedentary time (95% CI: 2.88 to 9.69) before adjustment to 1.76% (95% CI: −2.84 to 6.35) after adjustment for marital status, gender, education and Carstairs deprivation. The attenuation was mostly accounted for by area deprivation: in analyses only adjusting for gender, marital status, and education the difference between living in low and high crime neighbourhoods was still 4.51% higher sedentary time (95% CI: 1.02 to 8.02). For members of the LBC1936 in unadjusted analyses higher levels of natural space and poorer access to services, as indicated by SIMD, were associated with reduced sedentary time, and living at higher population densities associated with more sedentary time. However, these associations disappeared after adjusting for demographic and socioeconomic characteristics.

#### 3.2.2. Subjective Neighbourhood Measures

The associations between prospective subjective neighbourhood measures and sedentary time are shown in Table 5. In unadjusted analyses higher scores on perceived absence of services were associated with increased sedentary time in the Twenty-07 1950s retired cohort and the 1930s cohort. This remained significant after adjustment in the 1950s cohort and combined retired group. Additionally, for the 1950s retired cohort, there were significant associations, after adjustment, between sedentary time and fear of crime. Compared to those with no worries, “avoid” was associated with 4.66% (95% CI: 0.03 to 9.29) higher sedentary time while “never” was associated with 8.75% (95% CI: 2.12 to 15.38) higher sedentary time. Negative feelings about the area and reporting poorer social cohesion were also associated with increased sedentary time in unadjusted analyses. For the 1930s cohort, absence of goods and services apart, there were no further associations. Nor was there any evidence of a relationship between sedentary time and subjective neighbourhood measures for the 1950s employed cohort. The only measure available for LBC1936, neighbourhood attachment, was negatively associated with sedentary time but this was not significant in unadjusted (−0.20, 95% CI: −0.47 to 0.06, *p* = 0.17) or adjusted results (−0.20, 95% CI: −0.45 to 0.05, *p* = 0.12).

#### 3.2.3. Social Support Measures

Table 6 shows the results for unadjusted analyses between the social support measures and sedentary time. There was no evidence of any relationships for any of the measures in any of the cohorts.

#### 3.2.4. Social Participation Measures

The relationship between sedentary time and social participation measures for all cohorts are shown in Table 7. Being a carer was associated with reduced sedentary time for all retired people in the cohorts but not for the 1950s employed cohort. A significant relationship persisted after adjustment for the 1950s retired cohort (−3.36, 95% CI: −6.28 to −0.43) and combined retired group (−2.86, 95% CI: −4.67 to −1.04). While the coefficient was not significant for the 1930s cohort it was of comparable magnitude (−4.23, 95% CI: −9.95 to 1.50). Volunteering is also associated with reduced sedentary time for all cohorts, (weakest for the 1950s employed cohort), but is only significant in unadjusted analyses for the combined retired group (−2.42, 95% CI: −4.35 to −0.50), and was not significant (−1.79, 95% CI: −0.62 to 4.20) on adjustment. There were no associations between the number of sources of social contact and sedentary time for any of the cohorts.

The relationship between historic participation in social groups and sedentary time are shown in Table 7. Having been a member of a sports club, gym or exercise class was associated with reduced sedentary time for 1950s retired cohort even after adjustment (−4.42, 95% CI: −7.54 to −1.29), but not for the 1950s employed cohort. In unadjusted analyses, having been associated with a church or charitable organisation was associated with reduced sedentary time for the 1950s retired (−3.38, 95% CI: −6.65 to −0.12) and the equivalent coefficient for the 1950s employed cohort was of similar magnitude (albeit not significant) (−3.33, 95% CI: −8.54 to 1.87). This relationship was reduced on adjustment for socio demographic factors and no longer significant (−2.54, 95% CI: −5.32 to 0.25) even when the 1950s cohort retired and employed members were combined. There was no evidence that membership of educational or social groups were associated with sedentary time for the 1950s cohort. There was also no evidence that participation in any of the activities was associated with sedentary time for the 1930s cohort.

#### 3.2.5. Physical Home Environment

There is little evidence that the physical home environment is associated with sedentary time (see Table 8). While, in unadjusted analyses, living in a flat was associated with significantly increased sedentary time for the 1950s retired cohort (4.08, 95% CI: 0.12 to 8.05), and a similar but not significant coefficient was obtained for the 1930s cohort, this was dramatically reduced on adjusting for individual socioeconomic position and relationship status (0.57, 95% CI: −4.00 to 5.13) and no longer significant.

## 4. Discussion

The main finding of this study is that there were relatively few associations between sedentary time and most of the neighbourhood and social environment variables we investigated. We did find that crime rates were associated with increased sedentary time across all cohorts. This relationship persisted after adjustment for individual socioeconomic demographic factors but not area deprivation. Having had a fear of crime in the past did show an association with sedentary time after adjustment for retired members of the 1950s cohort. It is beyond the scope of this study to separate the effects of crime from other aspects of deprivation, but crime and fear of it may be an important determinant of sedentary time. Being a carer was associated with a modest reduction in sedentary time for all cohorts, the 1950s employed cohort excluded, and there were indications that volunteering had a similar influence albeit much weaker and not significant. The remaining significant associations after adjustment appear to be restricted to the 1950s retired cohort. Members of this cohort who were members of sports clubs or gyms, or church or charitable organisations in wave 5 were slightly less sedentary. In addition, past perceptions of perceived absence of shops and services were associated with increased sedentary time, unlike the current objectively reality. The comparative lack of significant results for the other cohorts is unlikely to be due to power alone as mostly the regression coefficients were small.

### 4.1. Comparisons with Literature

Our study includes the most comprehensive range of social and environmental measures in any study of the determinants of sedentary behaviour of which we are aware. The three studies [15,16,17] which have investigated environmental influences on objectively measured sedentary behaviour in older adults all assessed sedentary behaviour using ActiGraph monitors, as opposed to using devices with postural measures of sitting, and had results that are broadly consistent with our study. Van Holle et al. did not find an association between social cohesion and sedentary time [16]. They did however find a marginally significant interaction (*p* = 0.081), which might be a chance finding, between walkability and talking with neighbours for the prediction of sedentary time. Increased frequency of talking to neighbours was associated with reduced sedentary behaviour for people living in high walkability neighbourhoods, but showed no association with people living in low walkability neighbourhoods. However, the nature of this interaction was that neighbourhoods with low walkability had the lowest sedentary behaviour irrespective of participant’s status with respect to talking to neighbours. For the other two papers, social environmental measures played a somewhat more peripheral role to the main analyses. Sartini et al. [15] included social isolation as a possible determinant of diurnal patterns of sedentary behaviour and failed to find an association for this measure. This is consistent with the limited associations between the social participation and social support measures that we found. Van der Berg et al. [17] found an association between housing type and sedentary behaviour; however, their final model, unlike ours, did not adjust for other measures of socioeconomic position and their results may reflect housing type being a more general indicator of social circumstances rather than a person’s specific environment.

Given the scarcity of studies investigating determinants of objectively measured sedentary behaviour in older adults we have also compared our results to studies using self-reported sedentary behaviour or qualitative methodology. Our finding that providing care was associated with reduced sedentary behaviour is consistent with a qualitative study [13]. Our results are consistent with those of Van Cauwenberg et al. [19] who found that volunteering was associated with reduced self-reported TV viewing. However, given that we only find significant reductions in sedentary time for voluntering or past membership of church and charitable groups in selected unadjusted analyses, our support for Van Cauwenberg and co-workers’ results is somewhat weak.

Our finding that fear of crime is associated with increased sedentary time is consistent with a study from Belgium which found that feeling unsafe was associated with increased TV watching in adults over the age of sixty [19], however a study from Australia [22] failed to find an association between perceived daytime neighbourhood safety and TV viewing time. Our results are also consistent with those of Van Holle et al. [20] who found that perceived greater social cohesion and neighbourhood safety were associated with reduced sitting time at weekends among 55 to 65 year old Australian retirees, but not for the employed. Among the cohort we studied, the strongest associations between increased sedentary time on the one hand and fear of crime, social cohesion and poorer access to services on the other, were found for retired people. Similarly, the greatest reductions in sedentary time for past membership of sports clubs were found for the 1950s retired cohort. While Van Cauwenberg et al. [19] have shown that membership of social groups in general has been associated with reduced TV watching, our results suggest that protective effects of group membership may be confined to groups focused on specific activities such as sport.

The literature covering environmental influences on physical activity presents a similarly mixed picture. A systematic review of the influence of the built environment on physical activity found consistent evidence of associations between environmental influences and physical activity across age groups [50]. However, a systematic review focused only on older adults found limited evidence of a relationship between the physical environment and physical activity [51]. More recent studies suggest that the influence of the physical environment on physical activity varies for different groups. For example, crime has a larger impact on older adults than younger [52]. Overall, it is likely that different aspects of the environment may be relevant depending on how activity is measured [53].

The lack of significance for many of the other subjective neighbourhood and social measures is congruent with the lack of consistency within the broader empirical literature.

### 4.2. Implications

The associations between reduced sedentary behaviour and being a provider of care need to be investigated further. While we cannot infer causality from the association, caring for others may provide a purposeful and positive role within society and such may encourage behavioural change. However, we also need to be wary of the consequences of providing care. While providing care for grandchildren has been shown to be associated with good health [54], the strongest association (in terms of coefficients) was for the 1930s cohort and is likely to be driven by the need to provide care to partners. Such care is often very demanding and stressful and has the potential to have an adverse impact on health for older carers [55]. There is the potential that the relationship between providing care and reduced sedentary time does not represent a health promoting activity but additional detrimental physical demands.

That we find more evidence of determinants of sedentary behaviour for the retired people in the 1950s cohort is unlikely to be an issue of power, the coefficients for the 1930s cohort were also mostly smaller, and we found very few associations for LBC1936 which had a larger sample. The newly retired, or members of the third age, are less likely to have financial or health constraints than older age groups, and will have been freed from the constraints of employment [26]. This group are potentially the most susceptible to health interventions aimed at reducing sedentary behaviour.

### 4.3. Strengths and Limitations

The strengths of the study include the use of the activPAL3 monitor, which provides an objective measure of sedentary behaviour that correctly identifies posture. We have also achieved an extremely high data return rate (91%) compared to previous objective measure studies. The activPAL is also worn continuously, whereas other activity monitors are typically removed at night, and when showering or bathing, which introduces additional sources of error. The data covered an entire seven day period thus minimising any systematic variation over the course of the week. The activPAL3 monitor has only had direct validation in adults aged 18–65 years and children aged 6–17 years [30]. However, the previous activPAL model has been validated in older adults [56], and studies have shown that there is strong agreement between both models in both older adults [57] and in adults and children [58]. We are thus confident that the activPAL3 is valid for use in older adults.

By drawing from existing data sources, our study is the first that we are aware of to include a wide range of objective neighbourhood measures to investigate sedentary behaviour in older adults. However, the objective neighbourhood measures were operationalized using administrative areas and these may not accurately reflect the areas in which people live and we have made some compromises. For example while the walkability measure has been used in previous research [34] it only has two components dwelling density and intersection density. Measures of walkability typically contain four [37], the omitted sub-components being retail floor area ratio and land use mix. These were not relevant to the population for which this measure was originally derived [34] and were not available for this study. Using Global Positioning System (GPS) technology in combination with the activPAL monitor would have greatly strengthened our ability to identify the context in which sedentary behaviour occurred [59].

A wide range of self-reported measures of the neighbourhood environment, and social support and participation was used, however, in some cases comparable measures were not available for all cohorts. A consequence of the large number of measures is that some significant results might be due to chance findings, and this reinforces the need for our results to be taken in the context of the wider literature or replicated in other studies. Many of the self-report measures were recorded more than six years prior to the sedentary time measure. Participants’ perceptions of their social and physical environment may have changed in that time resulting in attenuated relationships.

For crime and access to services, we had both objective and subjective measures with the associations for the subjective measures being somewhat more robust to adjustment for socio demographic characteristics. This may be because of the limitations with regard to administrative areas (see above). Alternatively, it could be that characteristics of individuals may confound the relationship between sedentary behaviour and subjective perceptions of neighbourhood, or that less active people engage less with their environment and thus have a more limited view of the opportunities and services that are provided by that environment.

Data on volunteering and involvement in care provision were collected during the interviews in which participants were asked to wear the activPAL monitors and objective neighbourhood measures were linked to people’s postcode of residence at that time. The data used for the remaining variables were collected some years before (in a single wave for Twenty-07 in 2007/2008 and two waves for LBC1936 spanning the period form 2004 and 2010.) This may have influenced the strength of the relationship between measures and sedentary time. In addition, a small percentage of participants (which for Twenty-07 was 9%) will have changed residence in the time period between the interviews when the majority of subjective neighbourhood social environment measures were recorded and the start of Seniors USP.

We used percentage waking time spent sedentary as the outcome, it is the aspect of sedentary behaviour that has the strongest associations with health [6,60]. However, no single measure captures all aspects of sedentary behaviour [61], and there are alternative ways of conceptualising and operationalizing it [62] and one of these measures might be more strongly influenced by the neighbourhood and social environment.

Our study sample was drawn from pre-existing cohorts who were predominately urban and living in the central belt of Scotland and may not be generalizable to other areas.

The circumstances which led to members of the 1950s cohort retiring, such as poor health, may have altered the way in which the social and physical environment influences their sedentary behaviour.

Another weakness of our approach is the reliance on self-reports of sleep and waking times. Efforts to accurately identify sleep time from accelerometry data might prove fruitful for future research. A final limitation is that social and physical environment in which people live are complex systems [12] and there are limits to how well this can be modelled using traditional statistical methods and cohort data.

## 5. Conclusions

Our results add to the literature by indicating that, for retired older adults, being a carer may reduce sedentary behaviour. Overall, our results also suggest that the influences of the objective environment on sedentary behaviour are small and hard to detect. We provide some evidence that people who are at increased risk of crime, who have a fear of crime, or poor perceptions of their neighbourhoods, are likely to spend a greater proportion of their time sedentary. However, these results would also indicate that subjective perceptions of neighbourhoods have the strongest associations for people who are retired.

## Figures and Tables

**Table 1:** Number (n), Mean and Standard Deviation (SD) for sedentary time, continuous objective and subjective neighbourhood measures and social support measures by cohort and employment status.

	1950s Employed			1950s Retired			1930s Cohort			LBC1936		
	n	Mean	SD	n	Mean	SD	n	Mean	SD	n	Mean	SD
Percent Waking Time Sedentary (%)	110	58.3	11.2	200	62.2	10.3	119	68.2	10.9	271	62.5	10.4
*Objective neighbourhood measures*												
Natural Space (%)	101	59.9	13.2	182	58.7	14.6	102	58.9	12.9	262	55.2	15.1
SIMD Access (Fractional Rank)	110	0.46	0.28	200	0.45	0.27	119	0.43	0.27	271	0.41	0.25
SIMD Crime (Fractional Rank)	110	0.43	0.26	200	0.49	0.25	119	0.47	0.26	271	0.42	0.24
Walkability (One Unit Increase)	110	0.09	2.17	200	0.17	2.07	119	0.53	2.47	271	0.20	2.12
Pensioner Density (%)	110	17.4	7.37	200	17.2	6.40	119	19.8	6.98	271	19.9	7.19
Population Density (Persons/Hectare)	110	42.0	33.4	200	46.3	31.9	119	44.6	29.6	271	48.5	37.8
Carstairs (Fractional Rank)	110	0.39	0.29	200	0.44	0.31	119	0.47	0.32	271	0.30	0.25
Green Space at Output Area (%)	89	11.1	16.1	158	8.9	15.8	94	7.3	15.2	260	7.4	17.9
*Subjective neighbourhood measures*												
Social Cohesion	107	11.1	2.61	192	11.7	3.35	98	11.5	3.01			
Incivilities	107	2.67	2.28	182	4.04	3.32	105	2.97	2.94			
Absence Good and Services	106	1.22	1.38	192	1.60	1.45	109	1.33	1.43			
Physical Environment	109	1.38	1.10	197	1.80	1.55	113	1.68	1.48			
Attitude to Area	109	2.01	0.84	199	2.10	0.94	115	2.00	0.92			
Neighbourhood Attachment										269	22.5	4.76
*Social Support*												
Sources of Social Contact	109	3.77	0.56	199	3.77	0.58	118	3.75	0.60	253	3.77	0.54
n Sources Emotional Support	108	3.15	2.05	197	3.79	3.64	118	2.97	2.66			
n Practical Support	109	4.84	3.08	198	5.31	3.49	118	4.18	2.98			
Social Support										250	60.6	7.27
Perceived Support										269	20.0	2.00

**Table 2:** Categorical subjective neighbourhood, self-reported group membership and home environment variables for the Twenty-07 study.

	1950s: Employed		1950s: Retired		1930s Cohort	
	n	%	n	%	n	%
**Fear of crime (walking after dark)**						
No Worries	88	80.0	134	67.0	65	54.6
Uncomfortable	15	13.6	30	15.0	10	8.4
Try to Avoid	5	4.6	23	11.5	24	20.2
Never	1	0.9	11	5.5	15	12.6
Missing Data	1	0.9	2	1.0	5	4.2
*Membership of groups*						
**Church or Charitable**						
No	86	78.2	147	73.5	71	59.7
Yes ^1^	23	20.9	52	26.0	45	37.8
Missing Data	1	0.9	1	0.5	3	2.5
**Educational**						
No	97	88.2	184	92.0	93	78.2
Yes ^1^	12	10.9	15	7.5	23	19.3
Missing Data	1	0.9	1	0.5	3	2.5
**Social groups**						
No	103	93.6	174	87.0	81	68.1
Yes ^1^	6	5.5	25	12.5	35	29.4
Missing Data	1	0.9	1	0.5	3	2.5
**Sports club or gym**						
No	77	70.0	137	68.5	92	77.3
Yes ^1^	32	29.1	62	31.0	24	20.2
Missing Data	1	0.9	1	0.5	3	2.5
*Home environment*						
**Home Type**						
Detached	33	30.0	54	27.0	22	18.5
Semi-Detached	33	30.0	48	24.0	25	21.0
Terraced House	20	18.2	46	23.0	25	21.0
Any Flat	23	20.9	51	25.5	45	37.8
Missing Data	1	0.9	1	0.5	2	1.7
**Entrance on ground floor**						
No	10	9.1	26	13.0	27	22.7
Yes ^1^	100	90.9	173	86.5	92	77.3
Missing Data	0	0.0	1	0.5	0	0.0
**Internal stairs**						
One Level	24	21.8	48	24.0	47	39.5
With Stairs	86	78.2	151	75.5	72	60.5
Missing Data	0	0.0	1	0.5	0	0.0
**Own Garden**						
Other	11	10.0	33	16.5	34	28.6
Own Garden or Yard	98	89.1	166	83.0	81	68.1
Missing Data	1	0.9	1	0.5	4	3.4

^1^ Yes indicates a person who participated in an activity, while No indicates that they did not.

**Table 3:** Social participation and demographic characteristics for categorical variables available for the Twenty-07 and LBC1936 (Lothian Birth Cohort 1936) studies.

	1950s Employed		1950s Retired		1930s Cohort		LBC1936	
	n	%	n	%	n	%	n	%
**Volunteering**								
No	84	76.4	131	65.5	89	74.8	208	76.8
Yes ^1^	26	23.6	66	33.0	28	23.5	63	23.3
Missing Data	0	0.0	3	1.5	2	1.7	0	0.0
**Caring for others**								
No	66	60.0	108	54.0	102	85.7	183	67.5
Yes ^1^	44	40.0	91	45.5	17	14.3	87	32.1
Missing Data	0	0.0	1	0.5	0	0.0	1	0.4
**Educational Qualifications**								
No formal	10	9.1	15	7.5	34	28.6	36	13.3
Basic	65	59.1	96	48.0	61	51.4	133	49.1
Degree or Professional	35	31.8	89	44.5	24	20.2	102	37.6
**Gender**								
Male	70	63.6	75	37.5	54	45.5	140	51.7
Female	40	36.6	125	62.5	65	54.6	131	48.3
**Marital status**								
Married, Cohabiting	85	72.3	150	75.0	49	41.2	185	68.3
Single, Divorced, Separated	19	17.3	36	18.0	14	11.8	30	11.1
Widowed	5	5.5	14	7.0	56	47.1	56	20.7

^1^ Yes indicates a person who participated in an activity, while No indicates that they did not.

**Table 4:** Unadjusted and adjusted regression coefficients for the association between objective neighbourhood measures and sedentary time.

	1950s Employed	1950s Retired	1930s Cohort	LBC1936	Combined Retired Group ^1^
	β (95% CI)	β (95% CI)	β (95% CI)	β (95% CI)	β (95% CI)
Unadjusted					
**Natural Space Data Zone**					
1% Increase	0.5 (−1.20 to 2.21)	−0.18 (−1.22 to 0.86)	0.57 (−1.12 to 2.27)	−1.13 (−1.97 to −0.30) **	−0.57 (−1.17 to 0.04) ^+^
**Green Space Output Area**					
1% Increase	−0.75 (−2.21 to 0.7)	0.07 (−1.00 to 1.13)	0.09 (−1.33 to 1.52)	−0.08 (−0.80 to 0.64)	−0.01 (−0.56 to 0.53)
**Walkability**					
One Unit Increase	−0.51 (−1.48 to 0.47)	−0.19 (−0.89 to 0.51)	0.25 (−0.56 to 1.06)	0.32 (−0.27 to 0.90)	0.14 (−0.25 to 0.53)
**SIMD Access**					
SII	2.23 (−5.33 to 9.79)	−1.51 (−6.88 to 3.86)	−1.58 (−9.11 to 5.95)	−6.24 (−11.26 to −1.22) *	−3.50 (−6.78 to −0.22) *
**SIMD Crime**					
SII	0.12 (−7.97 to 8.21)	7.96 (2.23 to 13.70) **	4.18 (−3.6 to 11.95)	6.02 (0.92 to 11.11) *	6.29 (2.88 to 9.69) ***
**Pensioner density**					
1% Increase	1.41 (−1.46 to 4.28)	−1.88 (−4.13 to 0.36) ^+^	1.22 (−1.63 to 4.08)	−0.89 (−2.62 to 0.84)	−0.74 (−1.98 to 0.49)
**Population density**					
Person Hectares	−0.02 (−0.08 to 0.05)	0.01 (−0.04 to 0.06)	−0.01 (−0.08 to 0.06)	0.03 (0.00 to 0.06) ^+^	0.02 (−0.01 to 0.04)
Adjusting for Gender, Marital Status, Educational Qualifications, Area Deprivation					
**Natural Space Data Zone**					
10% Increase	0.71 (−1.08 to 2.51)	0.00 (−1.03 to 1.03)	0.77 (−1.01 to 2.56)	−0.71 (−1.59 to 0.18)	−0.21 (−0.82 to 0.41)
**Green space output area**					
10% Increase	−0.02 (−1.47 to 1.44)	−0.12 (−1.17 to 0.93)	0.00 (−1.45 to 1.44)	−0.03 (−0.72 to 0.66)	0.01 (−0.51 to 0.54)
**Walkability**					
One Unit Increase	−0.53 (−1.52 to 0.47)	−0.59 (−1.30 to 0.13)	0.00 (−0.88 to 0.88)	−0.07 (−0.69 to 0.56)	−0.23 (−0.64 to 0.18)
**SIMD Access**					
SII	2.64 (−5.95 to 11.23)	1.19 (−4.49 to 6.87)	2.46 (−5.85 to 10.77)	−4.47 (−9.69 to 0.75)	−0.95 (−4.41 to 2.52)
**SIMD Crime**					
SII	−3.19 (−14.39 to 8.01)	3.93 (−3.88 to 11.74)	−1.70 (−13.2 to 9.80)	2.59 (−4.08 to 9.26)	1.76 (−2.84 to 6.35)
**Pensioner Density**					
10% Increase	0.59 (−2.25 to 3.44)	−1.46 (−3.73 to 0.80)	1.03 (−1.92 to 3.98)	0.07 (−1.74 to 1.88)	−0.16 (−1.4 to 1.08)
**Population density**					
Person Hectares	−0.02 (−0.09 to 0.04)	−0.01 (−0.06 to 0.03)	−0.04 (−0.11 to 0.04)	0.01 (−0.02 to 0.05)	0.00 (−0.03 to 0.02)

^1^ Analyses adjusted for cohort; ^+^ *p* < 0.10, * *p* < 0.05, ** *p* < 0.01, *** *p* < 0.001.

**Table 5:** Unadjusted and adjusted regression coefficients for the association between subjective neighbourhood measures and sedentary time for the Twenty-07 cohorts.

	1950s Employed	1950 Retired	1930s Cohort	Combined Retired Group ^1^
	β (95% CI)	β (95% CI)	β (95% CI)	β (95% CI)
Unadjusted				
**Social Cohesion**				
One Unit Increase (Less Cohesion)	0.28 (−0.55 to 1.12)	0.50 (0.06 to 0.93) *	−0.13 (−0.89 to 0.63)	0.32 (−0.07 to 0.70)
**Incivilities**				
One Unit Increase	0.1 (−0.85 to 1.05)	0.33 (−0.11 to 0.78)	−0.49 (−1.23 to 0.25)	0.08 (−0.31 to 0.46)
**Absence of Goods and Services**				
One Unit Increase	0.12 (−1.45 to 1.69)	1.58 (0.61 to 2.55) **	1.32 (−0.14 to 2.78) ^+^	1.49 (0.68 to 2.29) ***
**Physical Environmental Problems**				
One Unit Increase	0.76 (−1.2 to 2.72)	0.63 (−0.30 to 1.57)	−0.58 (−1.98 to 0.81)	0.22 (−0.56 to 1.00)
**Fear of Crime-Walking at Night** (Ref = No Worries)				
Do It but Feel Uncomfortable		1.49 (−2.55 to 5.52)	−3.93 (−11.4 to 3.54)	−0.03 (−3.63 to 3.56)
Try to Avoid Doing It	Not estimated due to	4.21 (−0.30 to 8.72) ^+^	−0.24 (−5.49 to 5.01)	2.34 (−1.06 to 5.73)
Never Do It	low numbers	9.47 (3.20 to 15.73) **	0.14 (−6.15 to 6.44)	4.64 (0.27 to 9.01) *
**Feeling About Area**				
One Unit Increase	−0.15 (−2.62 to 2.31)	1.73 (0.56 to 2.90) **	−0.01 (−2.00 to 1.97)	1.22 (0.21 to 2.24) *
Adjusting for Gender, Marital Status, Educational Qualifications, Area Deprivation				
**Social Cohesion**				
One Unit Increase (Less Cohesion)	0.03 (−0.82 to 0.89)	0.33 (−0.13 to 0.79)	−0.37 (−1.18 to 0.43)	0.14 (−0.26 to 0.54)
**Incivilities**				
One Unit Increase	−0.13 (−1.07 to 0.82)	0.03 (−0.44 to 0.51)	−0.55 (−1.3 to 0.2)	−0.19 (−0.59 to 0.21)
**Absence of Goods and Services**				
One Unit Increase	0.55 (−1.17 to 2.28)	1.23 (0.17 to 2.29) *	0.84 (−0.69 to 2.38)	1.09 (0.22 to 1.96) *
**Physical Environmental Problems**				
One Unit Increase	0.73 (−1.21 to 2.66)	0.44 (−0.5 to 1.38)	−0.88 (−2.29 to 0.54)	0 (−0.79 to 0.78)
**Fear of Crime-Walking at Night** (Ref = No Worries)				
Do It but Feel Uncomfortable		1.86 (−2.27 to 6)	−5.11 (−12.78 to 2.55)	0.06 (−3.59 to 3.72)
Try to Avoid Doing It	Not estimated due to	4.66 (0.03 to 9.29) *	−1.42 (−7.12 to 4.27)	2.16 (−1.4 to 5.72)
Never Do It	low numbers	8.75 (2.12 to 15.38) *	−2.31 (−9.29 to 4.66)	3.29 (−1.36 to 7.94)
**Feeling about area**				
One Unit Increase	−0.61 (−3.19 to 1.96)	1.26 (0 to 2.52) ^+^	−0.96 (−3.25 to 1.34)	0.7 (−0.41 to 1.81)

^1^ Analyses adjusted for cohort. ^+^ *p* < 0.10, * *p* < 0.05, ** *p* < 0.01, *** *p* < 0.001.

**Table 6:** Regression coefficients for the relationship between sedentary time and social support measures for the Twenty-07 cohorts and LBC1936.

	1950s Employed	1950s Retired	1930s cohort	LBC1936	Combined Retired Group ^1^
	β (95% CI)	β (95% CI)	β (95% CI)	β (95% CI)	β (95% CI)
Emotional Support	−0.15 (−1.20 to 0.91)	0.10 (−0.30 to 0.50)	−0.54 (−1.28 to 0.21)		−0.05 (−0.41 to 0.3)
Practical Support	0.15 (−0.55 to 0.85)	−0.04 (−0.46 to 0.37)	−0.43 (−1.10 to 0.23)		−0.16 (−0.51 to 0.19)
Satisfaction with Social Support				0.14 (−0.04 to 0.31)	
Perceived Social Support				−0.51 (−1.14 to 0.11)	

^1^ Analyses adjusted for cohort.

**Table 7:** Unadjusted and adjusted regression coefficients for associations between sedentary time and social participation measures that are available for all cohorts.

	1950s Employed	1950s Retired	1930s Cohort	LBC 1936	Combined Retired Group ^1^
	β (95% CI)	β (95% CI)	β (95% CI)	β (95% CI)	β (95% CI)
Unadjusted					
**Volunteer** (No)					
Yes ^2^	−0.97 (−5.96 to 4.01)	−2.55 (−5.61 to 0.52)	−3.20 (−7.90 to 1.49)	−1.96 (−4.90 to 0.97)	−2.42 (−4.35 to −0.5) *
**Carer** (No)					
Yes ^2^	0.23 (−4.1 to 4.56)	−3.18 (−6.06 to −0.31) *	−3.83 (−9.48 to 1.81)	−2.89 (−5.53 to −0.25) *	−3.12 (−4.96 to −1.28) *
**Social Contact**					
Per Additional Source	−1.90 (−5.67 to 1.87)	−1.79 (−4.28 to 0.70)	0.97 (−2.35 to 4.30)	−1.04 (−3.41 to 1.34)	−0.85 (−2.37 to 0.67)
*Group Membership*					
**Church or Charitable** (No)					
Yes ^2^	−3.33 (−8.54 to 1.87)	−3.38 (−6.65 to −0.12) *	−0.87 (−5.00 to 3.26)		−2.33 (−4.88 to 0.21) ^+^
**Educational** (No)					
Yes ^2^	−3.9 (−10.70 to 2.89)	−1.33 (−6.82 to 4.16)	0.05 (−5.00 to 5.10)		−0.54 (−4.20 to 3.11)
**Social** (No)					
Yes ^2^	3.1 (−6.26 to 12.47)	0.24 (−4.14 to 4.61)	0.42 (−3.97 to 4.81)		0.33 (−2.72 to 3.39)
**Sports**					-
Yes ^2^	0.57 (−4.13 to 5.26)	−5.36 (−8.39 to −2.32) ***	−0.80 (−5.77 to 4.17)		3.95 (−6.56 to −1.34) **
Adjusting for Gender, Marital Status, Educational Qualifications, Area Deprivation					
**Volunteer** (No)					-
Yes ^2^	−0.74 (−5.71 to 4.23)	−2.18 (−5.30 to 0.93)	−2.15 (−7.14 to 2.83)	−1.09 (−3.95 to 1.78)	−1.77 (−3.69 to 0.15) ^+^
**Carer** (No)					
Yes ^2^	0.07 (−4.26 to 4.40)	−3.36 (−6.28 to −0.43) *	−4.23 (−9.95 to 1.50)	−2.28 (−4.84 to 0.29) ^+^	−2.86 (−4.67 to −1.04) *
**Social Contact**					
Per Additional Source	−0.94 (−4.80 to 292)	−1.39 (3.88 to 1.11)	0.64 (−2.77 to 4.05)	−0.48 (−2.85 to 1.89)	−0.43 (−1.93 to 1.08)
*Group Membership*					
**Church or Charitable** (No)					
Yes ^2^	−2.56 (−7.83 to 2.71)	−2.66 (−5.94 to 0.63)	0.58 (−4.00 to 5.17)		−1.65 (−4.27 to 0.97)
**Educational** (No)					
Yes ^2^	−5.47 (−12.32 to 1.37)	−0.57 (−6.08 to 4.93)	1.98 (−3.46 to 7.41)		0.44 (−3.32 to 4.21)
**Social** (No)					
Yes ^2^	1.25 (−8.07 to 10.56)	0.49 (−3.88 to 4.87)	0.08 (−4.31 to 4.46)		0.37 (−2.65 to 3.40)
**Sports** (No)					
Yes ^2^	1.97 (−2.67 to 6.60)	−4.42 (−7.54 to −1.29) **	0.04 (−5.07 to 5.15)		−2.72 (−5.39 to −0.05) *

^1^ Analyses adjusted for cohort. ^2^ Yes indicates a person who participated in an activity, while No indicates that they did not; ^+^ *p* < 0.10, * *p* < 0.05, ** *p* < 0.01, *** *p* < 0.001.

**Table 8:** Adjusted and unadjusted regression coefficients for the relationship between the home environment measures and sedentary time for the Twenty-07 study.

	1950s Employed	1950s Retired	1930s Cohort	Combined Retired Group ^1^
	β (95% CI)	β (95% CI)	β (95% CI)	β (95% CI)
Unadjusted				
Home Type (Detached)				
Semi-Detached	−0.55 (−5.99 to 4.90)	2.24 (−1.79 to 6.27)	1.62 (−4.71 to 7.94)	2.01 (−1.37 to 5.4)
Terraced house	−0.02 (−6.29 to 6.24)	3.51 (−0.57 to 7.58) ^+^	5.37 (−0.95 to 11.70) ^+^	4.15 (0.74 to 7.56) *
Any Flat	1.64 (−4.37 to 7.65)	4.08 (0.12 to 8.05) *	4.00 (−1.63 to 9.63)	4.00 (0.80 to 7.20) *
Floor of Entrance (Other)				
Ground	−3.76 (−11.10 to 3.57)	−3.49 (−7.76 to 0.78)	−0.17 (−4.92 to 4.58)	−1.90 (−5.05 to 1.25) *
Internal Stairs (Absent)				
Present	−0.34 (−5.47 to 4.79)	1.60 (−1.78 to 4.98)	−0.35 (−4.42 to 3.72)	0.75 (−1.83 to 3.33)
Private Garden (No)				
Yes	−2.09 (−9.18 to 5.00)	0.17 (−3.73 to 4.07)	−0.36 (−4.80 to 4.08)	−0.08 (−2.98 to 2.82)
Adjusting for Gender, Marital Status, Educational Qualifications, Area Deprivation				
Home type (Detached)				
Semi-Detached	1.03 (−4.64 to 6.70)	1.12 (−2.93 to 5.18)	−0.14 (−6.92 to 6.64)	0.54 (−2.92 to 4.00)
Terraced House	1.49 (−5.31 to 8.28)	1.61 (−2.65 to 5.88)	3.20 (−4.13 to 10.53)	1.96 (−1.70 to 5.62)
Any Flat	1.91 (−4.98 to 8.79)	0.57 (−4.00 to 5.13)	2.00 (−4.85 to 8.84)	1.01 (−2.67 to 4.70)
Floor of Entrance (Other)				
Ground	−3.19 (−10.66 to 4.27)	−0.38 (−5.00 to 4.22)	0.21 (−4.60 to 5.02)	0.00 (−3.25 to 3.24)
Internal stairs (Absent)				
Present	0.51 (−4.69 to 5.72)	3.32 (−0.15 to 6.79) ^+^	−0.06 (−4.21 to 4.08)	1.66 (−0.96 to 4.28)
Private Garden (No)				
Yes	−0.85 (−8.01 to 6.30)	2.59 (−1.48 to 6.66)	−0.57 (−5.10 to 3.96)	1.21 (−1.78 to 4.20)

^1^ Analyses adjusted for cohort; ^+^ *p* < 0.10, * *p* < 0.05.
